# Implementation of shared decision making by physician training to optimise hypertension treatment. Study protocol of a cluster-RCT

**DOI:** 10.1186/1471-2261-12-73

**Published:** 2012-09-11

**Authors:** Iris Tinsel, Anika Buchholz, Werner Vach, Achim Siegel, Thorsten Dürk, Andreas Loh, Angela Buchholz, Wilhelm Niebling, Karl-Georg Fischer

**Affiliations:** 1 Department of Medicine, Division of General Practice, University Medical Centre Freiburg, Elsässerstr. 2 m, Freiburg, 79110, Germany; 2 Clinical Trials Unit, University Medical Centre Freiburg, Elsässerstr. 2, Freiburg, 79110, Germany; 3 Institute of Medical Biometry and Medical Informatics, University Medical Centre Freiburg, Stefan-Meier-Str. 26, Freiburg, 79104, Germany; 4 Department of Medical Psychology, University Medical Centre Hamburg-Eppendorf, Martinistraße 52, Hamburg, 20246, Germany; 5 Department of Medicine, Division of Nephrology, University Medical Centre Freiburg, Hugstetterstr. 55, Freiburg, 79106, Germany

**Keywords:** Arterial hypertension, Cardiovascular diseases, Cardiovascular risk, Shared decision making, Educational training, Blood pressure control, Ambulatory blood pressure monitoring, Adherence, Primary care, Family medicine

## Abstract

**Background:**

Hypertension is one of the key factors causing cardiovascular diseases which make up the most frequent cause of death in industrialised nations. However about 60% of hypertensive patients in Germany treated with antihypertensives do not reach the recommended target blood pressure. The involvement of patients in medical decision making fulfils not only an ethical imperative but, furthermore, has the potential of higher treatment success. One concept to enhance the active role of patients is shared decision making. Until now there exists little information on the effects of shared decision making trainings for general practitioners on patient participation *and* on lowering blood pressure in hypertensive patients.

**Methods/Design:**

In a cluster-randomised controlled trial 1800 patients receiving antihypertensives will be screened with 24 h ambulatory blood pressure monitoring in their general practitioners’ practices. Only patients who have not reached their blood pressure target (approximately 1200) will remain in the study (T1 – T3). General practitioners of the intervention group will take part in a shared decision making-training after baseline assessment (T0). General practitioners of the control group will treat their patients as usual. Primary endpoints are change of systolic blood pressure and change of patients’ perceived participation. Secondary endpoints are changes of diastolic blood pressure, knowledge, medical adherence and cardiovascular risk. Data analysis will be performed with mixed effects models.

**Discussion:**

The hypothesis underlying this study is that shared decision making, realised by a shared decision making training for general practitioners, activates patients, facilitates patients’ empowerment and contributes to a better hypertension control. This study is the first one that tests this hypothesis with a (cluster-) randomised trial and a large sample size.

**Trial registration:**

WHO International Clinical Trials: http://apps.who.int/trialsearch/Trial.aspx?TrialID=DRKS00000125

## Background

Hypertension is one of the key factors causing cardiovascular diseases [1] which make up the most frequent cause of death in industrialised nations [2]. In spite of the high individual and societal burden of high blood pressure and cardiovascular disease respectively, about 60% of hypertensive patients in Germany treated with antihypertensives do not reach a target blood pressure of systolic / diastolic ≤ 140/90 mmHg in clinical measurement [3]. At the same time, scientific development and socio-cultural changes have initiated a change in medical decision making from a rather paternalistic towards a more participatory style in which both physician and patient play an active role. In Germany, patients are entitled to get fully informed on chances and risks of equivalent treatments [4]. In hypertension therapy, there is a considerable number of treatment alternatives, and patients want to be informed about options and take part in their treatment decisions [5].

One key concept to enhance patients’ active participation in medical decisions is shared decision making (SDM). It is characterised as an interactive process between physician and patient, acting according to the principle of equipoise. Both parties share information with the aim to build a consensus about the preferred treatment and its realisation. One precondition to realise SDM is the existence of equivalent treatment options [6,7].

It must be assumed that patients with hypertension who do not reach target blood pressure despite antihypertensive medication are – at least in their majority – not able to put behaviour change and / or medical adherence into practice. In these cases, first of all, general practitioners (GP) should support patients to improve their health behaviour [8-10].

Evidence on SDM interventions showed positive effects of SDM on patients’ participation, satisfaction with their treatment, decisional conflict, knowledge about the disease, adherence, and partly clinical outcomes [11-17]. Nevertheless, study designs, intervention programmes and measurement instruments vary considerably, sample sizes are often small and results are inconsistent.

Within the research programme “The patient as partner in medical decision making”, funded from 2001 by the German Ministry of Health, an SDM training programme for clinicians has been developed [6] and evaluated in various studies [14,18].

Until now there exists little information on the effects of SDM trainings for GPs on patient participation *and* clinical outcomes [19]. This holds also for blood pressure level in antihypertensive treatment where only one study (non-randomised and with a comparably small sample size) has been conducted until now [17]. The aim of our study is therefore to implement and evaluate an SDM training programme for GPs within the context of hypertension treatment in primary care.

### Research objectives

Our study attempts to answer the following research questions: Does the SDM training programme for GPs

1) enhance patients’ perceived participation?

2) optimise the blood pressure values of patients?

3) enhance patients’ knowledge about hypertension?

4) improve patient adherence?

## Methods/Design

The Ethical Committee of the University Medical Centre Freiburg approved this study on 26 February 2009. A non-substantial protocol amendment was approved on 7 March 2012 by the Ethical Committee of the University Medical Centre Freiburg.

### Study design

**Figure 1:**
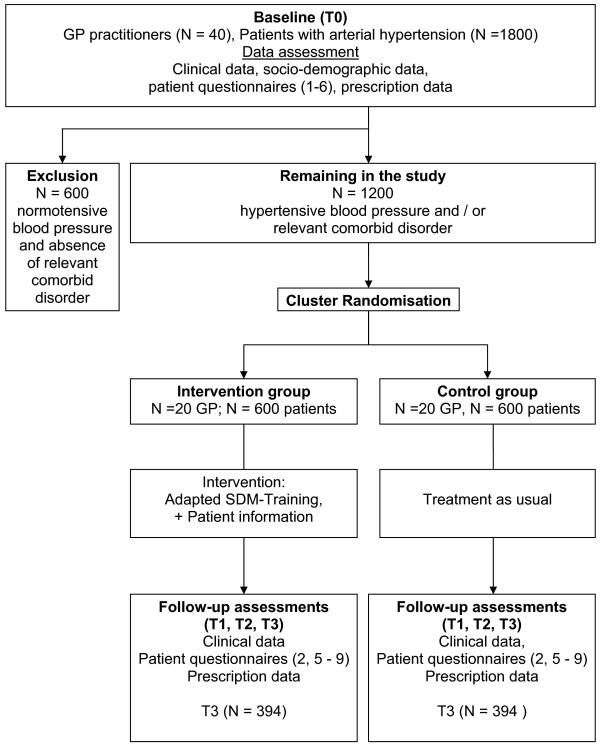
Study protocol diagram.

The study will be conducted as a cluster-RCT with 40 GP practices in the Suedbaden region, Germany. Patients who receive an antihypertensive medication will be invited to take part in the study. After a baseline data assessment (T0), GPs will be randomised into intervention group (SDM training and dissemination of printed patient information on hypertension and antihypertensive treatment) and into control group (no training, treatment as usual). After the intervention, three follow-up data assessments are scheduled (T1, T2 and T3; cf. Figure 1).

### Study population

#### General practitioners

About 120 GP practices of a network of accredited general practitioners and academic GP practices associated with the Department of General Practice of the University Medical Centre Freiburg (Germany) will be informed about the study.

#### Patients

Each participating GP is asked to include 30 patients in the study who are currently treated for hypertension. These patients have to fulfil the following criteria.

Inclusion criteria: (1) Repeated prescription of antihypertensive medication, (2) at least 18 years old, (3) insured by a statutory health fund, (4) understanding of the German language.

Exclusion criteria: Dementia, mental handicap, or short life expectancy

#### Recruitment procedure

To assure a random sample of the patients in each GP practice, medical assistants of the practices (MA) will be instructed to invite every third consecutive patient fulfilling the inclusion criteria to take part in the study. For all patients fulfilling the inclusion criteria and willing to take part in the study, a baseline assessment is performed. This includes ambulatory blood pressure measurement (ABPM) to screen blood pressure control in all recruited patients, further clinical data and a patient questionnaire. Recruiting takes place over half a year.

After baseline ABPM, patients will be classified as ‘controlled’ treated if their blood pressure values are below the thresholds for hypertension measured by ABPM: mean values for 24 h ≤ 130/80 mmHg, ≤ 135/85 mmHg during the day, and ≤ 120/70 mmHg at night [1,20]. These patients with ‘controlled’ hypertension will be excluded from the study if none of the following comorbid disorders are diagnosed: diabetes mellitus, coronary heart disease / heart attack, stroke / transient ischaemic attack (TIA) or peripheral arterial occlusive disease (PAOD). Patients with ‘uncontrolled’ arterial hypertension – i.e. one of the thresholds mentioned above is exceeded – remain in the study. This holds as well for enrolled patients with any of the above-mentioned comorbid diagnoses, irrespective of their blood pressure values.

### Randomisation of the GP practices

As soon as the baseline assessment has been completed, GP practices will be randomised into intervention and control group. The randomisation will be performed by lot procedure. Thus, about half of the GPs will be assigned to the intervention and half of the GPs to the control group.

### Intervention

GPs of the intervention group will take part in an evaluated SDM training [6]. The training programme will be adapted to the requirements of hypertension treatment in general practice. The training involves at least two of three training sessions, with each session lasting three hours. The programme includes the following elements: (1) Information on arterial hypertension (epidemiology, treatment, patient adherence etc.), (2) principles of physician-patient-communication and risk communication, (3) information on the SDM concept including the implementation of the steps of the SDM process [7,10], (4) integration of Motivational Interviewing to enhance the patients’ intrinsic motivation to change health behaviour [8,9], (5) introduction of a decision table listing various options to lower cardiovascular risk, and (6) use of case vignettes for role plays simulating physician-patient consultations.

After the SDM training, GP practices will receive printed patient information developed by the German College of General Practitioners and Family Physicians (DEGAM) regarding blood pressure and the role of physical activity, healthy nutrition, and smoking cessation. GPs will be appealed to deliver patient information to the patients with hypertension.

GPs of the control group treat their patients as usual. They are invited to take part in the SDM training after the study.

### Data assessment and instruments

#### Clinical data

(1) Systolic and diastolic office blood pressure measurement in mmHg, (2) comorbidity: diabetes mellitus (and HbA1c), coronary heart disease / heart attack, ischaemic stroke / transient ischaemic attack or peripheral arterial occlusive disease, (3) cholesterol: total, HDL and LDL cholesterol, (4) 24 h ambulatory blood pressure monitoring (ABPM), (5) cardiovascular 10-year-risk score calculated according to the algorithm of the cardiovascular risk calculator *arriba* (http://www.arriba-hausarzt.de/arriba/index.html).

#### Patient self-reporting instruments

(1) Index reflecting patients’ intention to treat hypertension [12], (2) Medication Adherence Report Scale (MARS-D) [21], (3) helping relationship in taking medication [22], (4) Autonomy Preference Index (API) [23], (5) Trust in Physician Scale (TPS) [24], (6) knowledge about hypertension (8 items; developed by our research group), (7) medical decisions which are made with the GP during the consultation when ABPM results are reviewed, (8) patients’ perceived participation in medical decision making (SDM-Q-9) [25], (9) utilisation of further health care services.

#### Prescription data

(1) Current medication prescription plans of the GP practices (before each ABPM) including data on medication to lower blood pressure and cholesterol (ATC codes C02-C04 and C07–C10), dose and number of each entity, and time of day when a medication is taken. (2) Data of filled prescriptions (ATC codes C02-C04 and C07–C10). The prescription data will be used to define a medication possession ratio (MPR) [26] for each patient between the beginning of 2009 and the end of 2010. This feasibility subproject will be blinded for the study arm status of the patient.

All used scales – except knowledge questions and utilisation of further health care services – have been validated by other authors and translated into German by a consensus procedure. Retranslations (from German into English) were authorised by the original author. (The only exception is the Trust in Physician Scale because we could not get in contact with its authors.)

#### Socio-demographic and other data

Besides age, gender, degree of education, employment status and family history of cardiovascular events we ask whether patients are living with a partner. Furthermore height and weight as well as tobacco consumption will be assessed.

### Study endpoints

Co-primary endpoints are (1) change of patients’ perceived participation (measured by SDM-Q-9) from T0 to T1, T2 and T3, evaluated by the mean effect over the three time points, and (2) change of systolic blood pressure (mean of 24 h), measured by ABPM [27], from T1 to T2 and T3, evaluated by the mean effect over the two follow-up time points. While the intervention may affect perceived participation directly at the first visit after intervention (T1), changes in blood pressure cannot be affected before the second visit (T2).

Secondary endpoints are (1) change of diastolic blood pressure (24 h mean), (2) change of knowledge score, (3) change of adherence, measured by MARS-D and the MPR, and (4) change of cardiovascular risk score (CVR), all evaluated by the mean effect over the two and three follow-up time points, respectively. While the intervention may affect knowledge directly after T0, the other secondary endpoints will be affected at T2 at the earliest.

#### Hypotheses on predictive factors

The study should also be used to investigate whether patient characteristics like API, CVR, MARS-D, Intention-To-Treat-Hypertension-Index, helping relationship in taking medication and high knowledge score may predict treatment effects.

### Study procedure

In all participating GP practices identical ABPM instruments will be installed by supervised personnel. Medical assistants (MAs) of GPs will be instructed about utilisation of the ABPM, recruitment procedure and data collection.

Patients who agree to take part in the study sign an informed consent form. Blood pressure, gender, age and comorbidity of patients who do not agree to take part in the study will be anonymously assessed to account for a possible selection bias due to the study enrolment.

#### Baseline assessment

The baseline assessment includes all clinical data, socio-demographic data and prescription data. Before the ABPM will be performed, patients complete a first questionnaire including socio-demographic data, intention-to-treat-hypertension index [12], medication adherence report scale (MARS-D) [21], helping relationship in taking medication [22], Autonomy Preference Index (API) [23], Trust in Physician Scale (TPS) [24], and knowledge about hypertension (8 items; developed by our research group).

If the average blood pressure in the ABPM protocol exceeds 130/80 mmHg (24 h mean) or 135/85 mmHg (daytime mean) or 120/70 mmHg (night mean), previous blood pressure treatment is considered as ‘uncontrolled’ [1,20,28]. These patients – as well as patients with any of the above-mentioned comorbidities – remain in the study. In the following visit GPs discuss with these patients possible consequences regarding the subsequent treatment. Immediately after this baseline consultation, patients will receive a second questionnaire including the above-mentioned self-reporting instruments ‘treatment decision made to control hypertension’ (instrument 7) and SDM-Q-9 (instrument 8).

#### Follow-up assessments T1, T2, and T3

The follow-up assessments take place every 6 (+ − 2) months. In each follow-up, all clinical data and prescription plans will be assessed. After ABPM and the following consultation focusing blood pressure protocol, patients are asked to complete a follow-up questionnaire which includes MARS, TPS, knowledge about hypertension, treatment decision made to control hypertension, SDM-Q-9, tobacco consumption, height and weight, and utilisation of further health services.

#### Incentives reliant on realisation of data assessment

The GP practices or medical assistants, respectively receive a case payment of 10 € per complete dataset of each patient in each assessment. The ABPM equipment will be offered to the GPs after the end of the study.

### Data management

All pseudonymised data of the patient questionnaires, forms, prescription plans, ABPM protocols and filled prescriptions will be merged into an SPSS data file. The quality of the data entry of each assessment will be proved by a 10% random sample check. Additionally, extensive plausibility checks are performed.

Major analyses will be conducted by the Clinical Trials Unit at the University Medical Centre Freiburg. Statistical programming will be performed with the Statistical Analysis System (SAS) Version 9.2 or higher.

### Sample size calculation

The sample size calculation resulted in about 40 practices (20 per intervention group) with a total of 1200 patients (600 per treatment group), including about 20% drop outs on patient as well as practice level. Hence, data on 788 patients are expected to be available for analysis (i.e. about 33 practices with 24 patients each). Studies regarding hypertension prevalence [3,29] assume that about 1/3 of recruited patients attain blood pressure control. Therefore 1800 patients should be involved into the baseline data assessment. Thereof 2/3 or 1200 patients respectively will remain in the study.

Due to changes in the statistical analysis plan, a power calculation for the new analysis strategy has been performed (see section ‘Power calculation for the analysis of primary endpoints’).

### Statistical analyses

#### Analysis principle

The analysis of the primary endpoints ‘change of perceived participation’ and ‘change of systolic blood pressure will be done according to the intention-to-treat (ITT) principle. As we have no means to define protocol violations on the patient level and there were made no attempts to assess compliance at the practice level, no per-protocol analyses are planned.

#### Investigation of missing pattern

Prior to the analysis, the missing patterns will be explored. Depending on the findings, strategies will be worked out to reduce missing values. Possible strategies are, for example, imputation or exclusion of single items with many missing values in questionnaires.

In addition, we will analyse the missing patterns with respect to frequency and association with patient characteristics, with outcome measurements at other time points and with treatment status. The overall aim is to provide arguments to justify the use of a mixed model analysis, which formally relies on the missing at random (MAR) assumption.

#### Analysis of primary and secondary endpoints

For illustration of the main trends, boxplots will be used to visualise the distribution of primary and secondary outcome measurements at baseline and follow-up measurements as well as changes from baseline. In addition, the changes in the primary outcomes SDM and systolic blood pressure are plotted for each patient separately to explore the individual course of measurements.

The analysis of effectiveness of the intervention on the primary endpoints ‘change of perceived participation’ and change of systolic blood pressure will be performed with a mixed effects model of type

(1) $$Y_{\mathrm{ijk}}=\mu +\lambda Y_{\mathrm{ijb}}+a_{i}+\beta_{k}I_{i}+\gamma_{\mathrm{ij}}+\delta_{k}\epsilon_{\mathrm{ijk}}$$

where index *i* specifies the practice, *j* the patients (*j* = *1,…, no. of study patients in the corresponding practice)* and *k* the time point (*k = 2, 3* for systolic blood pressure and *k = 1,2,3* for perceived participation). In addition, μ is the intercept, α_i_ the random practice effect, β_k_ the fixed effect of intervention at time point T_*k*_, γ_ij_ the random patient effect, δ_k_ the fixed effect for time point T_*k*_, λ the fixed effect of baseline measurement Y_*ijb*_ (with *b = 0* for perceived participation and *b = 1* for systolic blood pressure) and ϵ_ijk_ the error term.

This model accounts for the variation within and between the clusters (medical practices). The effect of intervention (β) will be tested based on the mean of estimated effects over the different follow-up time points, i.e. $\beta =\frac{1}{2}\left(\beta_{2}+\beta_{3}\right)$ for systolic blood pressure and $\beta =\frac{1}{3}\left((\beta_{1}+\beta_{2}+\beta_{3}\right)$ for perceived participation. To adjust for multiple testing of the (co-) primary endpoints, a Bonferroni correction of the (two-sided) significance level α =0.05 will be applied, resulting in α* = α/2 = 0.025.

According to the EMA points to consider adjustment for baseline covariates [30] in ‘change from baseline’ analyses, the baseline value of the respective endpoint will be adjusted for.

The secondary endpoints will be analysed analogously with significance level 5%. No adjustment for multiple testing will be used.

For the endpoints systolic and diastolic blood pressure, medication adherence report scale (MARS) and cardiovascular risk score (CVR) with baseline T1, a model with baseline T0 is also considered as a sensitivity analysis.

For the primary endpoints, an additional sensitivity analysis based on a model adjusted for the prognostic factors practice, age, gender, baseline values of cardiovascular risk and comorbidity, MARS, API, intention to treat hypertension, helping relationship in taking medication, knowledge about hypertension, TPS and an indicator for treatment outside medical practice (aggregated over the complete study period) is conducted.

As an exploratory analysis, the influence of the prognostic factors mentioned above on the primary endpoints at baseline will be investigated.

No interim analysis will be performed during the course of the study.

#### Analysis of hypotheses on predictive factors

The hypotheses on the predictiveness of the factors API, CVR, intention to treat hypertension, helping relationship in taking medication, adherence, and knowledge will be addressed visually by depicting the mean values of the change in the two primary outcomes stratified by the factors in three (API) and two groups (all other factors) based on the terciles and median, respectively. In addition, regression models will be fitted in analogy to the primary analyses that are assuming identical effects at each time point, but allowing an interaction with the factor of interest, i.e. intervention. Since the influence of API on the change in perceived participation is expected to be strongest for medium sized API and less pronounced for small and large values [23,31], a quadratic transformation of API and an interaction between the intervention and this term is included in the model when considering API. Final statements on the predictiveness will be based on the significance of the interactions.

#### Additional analyses

Further analyses will be performed: (1) Association of practice characteristics with practice specific treatment effects, (2) predictiveness of change in a) perceived participation and b) TPS on change in systolic blood pressure, (3) association between a) change in perceived participation and change in TPS and b) change in adherence and change in perceived participation and systolic blood pressure, (4) comparison of the MPR with patients’ self-assessed adherence (MARS).

#### Power calculation for the analysis of primary endpoints

For the power calculation we assume that 788 patients are available for analysis with 24 patients per practice (i.e. about 33 practices) according to the original sample size calculation (see above, section ’sample size calculation’). Since Kerry and Bland [32] state that the intra-cluster correlation in studies where the intervention is aimed at changing the doctor’s behaviour may be larger than in other cluster-randomised studies, we calculate the power assuming an intra-cluster correlation (ICC) of 0.05 and 0.03 which leads to a variance inflation factor [33] of 2.15 and 1.69, respectively. Hence, the sample size 788 in the cluster-randomised trial would correspond to a sample size of 366 and 466 patients, respectively, in a study with individual randomisation.

Since knowledge on the expected effects for systolic blood pressure and perceived participation is rare, we base the calculation on the standardised effect size [34]. According to Machin and Campbell (2005) [35], a standardised effect size of 0.2 and 0.5 corresponds to a small and moderate effect of the intervention, respectively. Under the reasonable assumption of a standardised effect size of 0.3 or 0.35, a conservative estimate of the ICC (0.05) and a significance level of α = 2.5% for the test on either co-primary endpoint, i.e. a global significance level of 5%, the power is about 74% and 87%, respectively. To account for the uncertainty in both the effect size and ICC, we use several choices of these parameters to get an overview of possible settings. Depending on the specific parameter setting, the power varies between 37% and 100% as displayed in Table 1. 

**Table 1:** Power of analysis of primary endpoints under different assumptions of the intra-cluster correlation coefficient (ICC) and the standardised effect size

**Standardised effect size**	0,20	0,25	0,30	0,35	0,40	0,50	0,20	0,25	0,30	0,35	0,40	0,50
**ICC**	0,05	0,05	0,05	0,05	0,05	0,05	0,03	0,03	0,03	0,03	0,03	0,03
**Power (in %)**	37,14	55,96	73,51	86,58	94,35	99,45	46,71	67,62	84,05	93,78	98,11	99,92

These calculations are based on the assumption of only one measurement per patient during follow-up. They can be considered as conservative for the situation of repeated measurements at T1, T2, and T3, since this gives additional information (s. Table 1).

## Ethics and data protection

A specific data protection concept will be established in cooperation with the data protection commissioner of the University Medical Centre Freiburg. Patient data will be pseudonymised prior to the analyses. The data privacy protection according to § 75 SGB X will be fulfilled. All institutions cooperating in the study agree with the data protection concept before any data transfer will be realised.

Patients who agree to take part in the study sign an informed consent form approved by the Ethical Committee and the data protection commissioner of the University Medical Centre Freiburg.

All patient questionnaires will be sealed by patients themselves and forwarded to the responsible medical assistant within the GP practice, and neither medical assistants nor GPs will have an insight into questionnaires that have been completed by patients.

## Discussion

Cardiovascular diseases and resulting morbidity and premature death can be reduced by increasing hypertension control. While effective medication and a broad knowledge about health behaviour change to lower blood pressure exist, many patients have difficulties with medication adherence and integrating lifestyle changes into their everyday life. The hypothesis underlying this study is that shared decision making realised by SDM training for practitioners activates patients, facilitates patients’ empowerment and contributes to a better hypertension control. This study is the first one that tests this hypothesis with a (cluster-) randomised trial and a large sample size.

## Abbreviations

ABPM:Ambulatory blood pressure monitoring / measurement; API:Autonomy Preference Index; ATC:Anatomical Therapeutic Chemical Classification System; BP:Blood pressure; CVR:Cardiovascular risk; DEGAM:German College of General Practitioners and Family Physicians; DFG:German Research Foundation; GP:General Practitioners; ICC:Intra-cluster correlation; ITT:Intention-to-treat; MAR:Missing at random; MARS-D:Medication Adherence Report Scale; MA:Medical assistants of the practices; MPR:Medication possession ratio; PAOD:Peripheral arterial occlusive disease; RCT:Randomised Controlled Trial; SDM:Shared decision making; SDM-Q-9:9-item-questionnaire measuring patients’ perceived participation in medical decision making; TIA:Transient ischaemic attack; TPS:Trust in Physician Scale.

## Competing interests

The authors declare that they have no competing interests.

## Authors’ contribution

KGF, WN, AL, TD, IT conceived the study. AB, WV, IT and AB planned the statistical analysis. AS and all other authors have been involved in drafting the manuscript and they revised it critically. All authors read and approved the final manuscript.

## Pre-publication history

The pre-publication history for this paper can be accessed here:

http://www.biomedcentral.com/1471-2261/12/73/prepub
